# Does pulse oximeter use impact health outcomes? A systematic review

**DOI:** 10.1136/archdischild-2015-309638

**Published:** 2015-12-23

**Authors:** Abigail J Enoch, Mike English, Sasha Shepperd

**Affiliations:** 1 Nuffield Department of Population Health, University of Oxford, Oxford, UK; 2 KEMRI-Wellcome Trust Research Programme, Nairobi, Kenya; 3 Nuffield Department of Medicine, University of Oxford, Oxford, UK

**Keywords:** Health services research, Outcomes research, Paediatric Practice, Respiratory, Evidence Based Medicine

## Abstract

**Objective:**

Do newborns, children and adolescents up to 19 years have lower mortality rates, lower morbidity and shorter length of stay in health facilities where pulse oximeters are used to inform diagnosis and treatment (excluding surgical care) compared with health facilities where pulse oximeters are not used?

**Design:**

Studies were obtained for this systematic literature review by systematically searching the Database of Abstracts of Reviews of Effects, Cochrane, Medion, PubMed, Web of Science, Embase, Global Health, CINAHL, WHO Global Health Library, international health organisation and NGO websites, and study references.

**Patients:**

Children 0–19 years presenting for the first time to hospitals, emergency departments or primary care facilities.

**Interventions:**

Included studies compared outcomes where pulse oximeters were used for diagnosis and/or management, with outcomes where pulse oximeters were not used. Main outcome measures: mortality, morbidity, length of stay, and treatment and management changes.

**Results:**

The evidence is low quality and hypoxaemia definitions varied across studies, but the evidence suggests pulse oximeter use with children can reduce mortality rates (when combined with improved oxygen administration) and length of emergency department stay, increase admission of children with previously unrecognised hypoxaemia, and change physicians’ decisions on illness severity, diagnosis and treatment. Pulse oximeter use generally increased resource utilisation.

**Conclusions:**

As international organisations are investing in programmes to increase pulse oximeter use in low-income settings, more research is needed on the optimal use of pulse oximeters (eg, appropriate oxygen saturation thresholds), and how pulse oximeter use affects referral and admission rates, length of stay, resource utilisation and health outcomes.

**What is already known on this topic**

- Hypoxaemia is a common complication of pneumonia, bronchiolitis, asthma and sepsis and is associated with increased risk of death in children.
- Pulse oximeters are a low-cost intervention that could help reduce child mortality by more effectively diagnosing and monitoring children with hypoxaemia.
- Pulse oximeters are often not available or not used in low-income settings, but several international projects aim to increase their availability and use.

**What this study adds**

- The evidence, while low quality, suggests pulse oximeter use may improve children's mortality rates, morbidity measurement, hospital length of stay and admission of hypoxic children.
- In the included studies, pulse oximeters were often important for physician's clinical decision-making about children's treatment and management, and their use generally increased resource utilisation.
- More research is needed on optimal thresholds to use for hypoxaemia definitions, and on how pulse oximeter use affects resource utilisation and impacts health outcomes.

## Introduction

In newborns, children and adolescents hypoxaemia is associated with increased risk of death, and is a common complication of bronchiolitis, pneumonia, asthma and other serious conditions (eg, sepsis).^1–4^ Pulse oximetry is a low-cost intervention that could reduce child mortality, in line with Millennium Development Goal 4, by enabling early detection of hypoxaemia and improving accurate diagnosis, thereby increasing the chance of prompt, effective treatment.

Despite the potential to improve health outcomes, pulse oximeters are often not available, particularly in low-income settings. For example, only 38% of Nigerian tertiary hospitals and 3 of 22 Kenyan hospitals providing physician internship training had pulse oximeters in 2011 and 2012, respectively.^5^ ^6^ To promote access, pulse oximeters have been designed for low-income settings, for example, Lifebox, a low-cost, robust, portable, battery-operated oximeter.^7^ Other designs deliver pulse oximeter results to smartphones, using their spread to remote areas.^8^ Initiatives supporting pulse oximeter uptake include the WHO's Global Pulse Oximetry Project, Lifebox donations, and the BMJ Christmas Appeal.^7^ ^9^ ^10^

Evidence suggests that pulse oximeters identify 20–30% additional hypoxic children compared with using clinical signs alone, for example, grunting and depressed consciousness, which can be imprecise.^1^ ^11^ However, evidence of an association between hypoxaemia and mortality is not necessarily evidence that pulse oximetry implementation improves outcomes, particularly taking a broad health system perspective, when health worker actions, characteristics of children and health facilities, and additional resources, all interact to impact outcomes.

In the complex world of health systems, pulse oximetry could lead to improved health outcomes and system efficiencies, and reduced resource use, by helping health workers promptly diagnose children and initiate treatment, and by improving diagnostic accuracy, thereby preventing unnecessary admissions and treatments. Alternatively, pulse oximetry could lead to unnecessary admissions, treatment, referrals, and/or discharge delays, if thresholds for admission, referral or intervention are inappropriate.

As pulse oximetry availability increases at primary and community care levels in low-income countries, understanding the health system implications is increasingly important, particularly how pulse oximetry impacts resource utilisation. In high-income countries, guidance for routine screening with pulse oximetry is inconsistent, with some suggesting it is unhelpful.^12–17^ Debate also remains about optimum hypoxaemia definitions, especially at altitude.^2^ ^18–22^

We therefore reviewed the evidence on how pulse oximetry introduction impacts health and service use outcomes.

## Methods

We addressed the question “Do newborns, children and adolescents aged up to 19 years have lower mortality rates, lower morbidity, and shorter length of stay where pulse oximeters are used to inform diagnosis and treatment (excluding operative surgical care) compared with where pulse oximeters are not used?” Our secondary research question was, “What proportion of newborns, children and adolescents are given oxygen therapy where pulse oximeters are used compared with where pulse oximeters are not used.”

Studies were included if they recruited newborns, children and/or adolescents aged up to 19 years, presenting for the first time to a hospital, emergency department (ED), or primary care facility, regardless of setting. Studies assessing pulse oximeters in screening healthy newborns before discharge, or monitoring, for example, during surgery were excluded.

We included studies with at least one intervention group in which a pulse oximeter reading was taken and at least one control group in which pulse oximetry was not used. We included studies reporting mortality, morbidity (illness severity, ie, pneumonia severity scores, disability at discharge) and length of stay. Descriptive studies were excluded.

We systematically searched the Database of Abstracts of Reviews of Effects, Cochrane, Medion, PubMed, Web of Science, Embase, Global Health, CINAHL and WHO Global Health Library, with no language restrictions (Search terms—see online supplementary appendix I). Study references were checked. Websites of non-governmental organisations, health organisations and development organisations were searched for unpublished reports using ‘pulse oximeter’ and ‘pulse oximetry’. Topic experts were contacted for additional materials.

Studies that were not relevant based on title/abstract were excluded. We read the remaining studies’ full texts and excluded those not fulfilling the inclusion criteria. All full texts were read by a second person, and if inclusion was uncertain, a third. We extracted data using a tailored Cochrane data collection form and assessed risk of bias using the Cochrane ACROBAT tool.^23^ ^24^

We intended to calculate risk ratios, mean differences and CI’s, and if possible pool data within subgroups and conduct a meta-analysis. However, due to the small number of studies and the study design/outcome variability this was not possible. Instead we narratively describe the evidence using a structured approach while drawing insights where possible, a standard strategy in such situations.

## Results

### Search results

**Figure 1:**
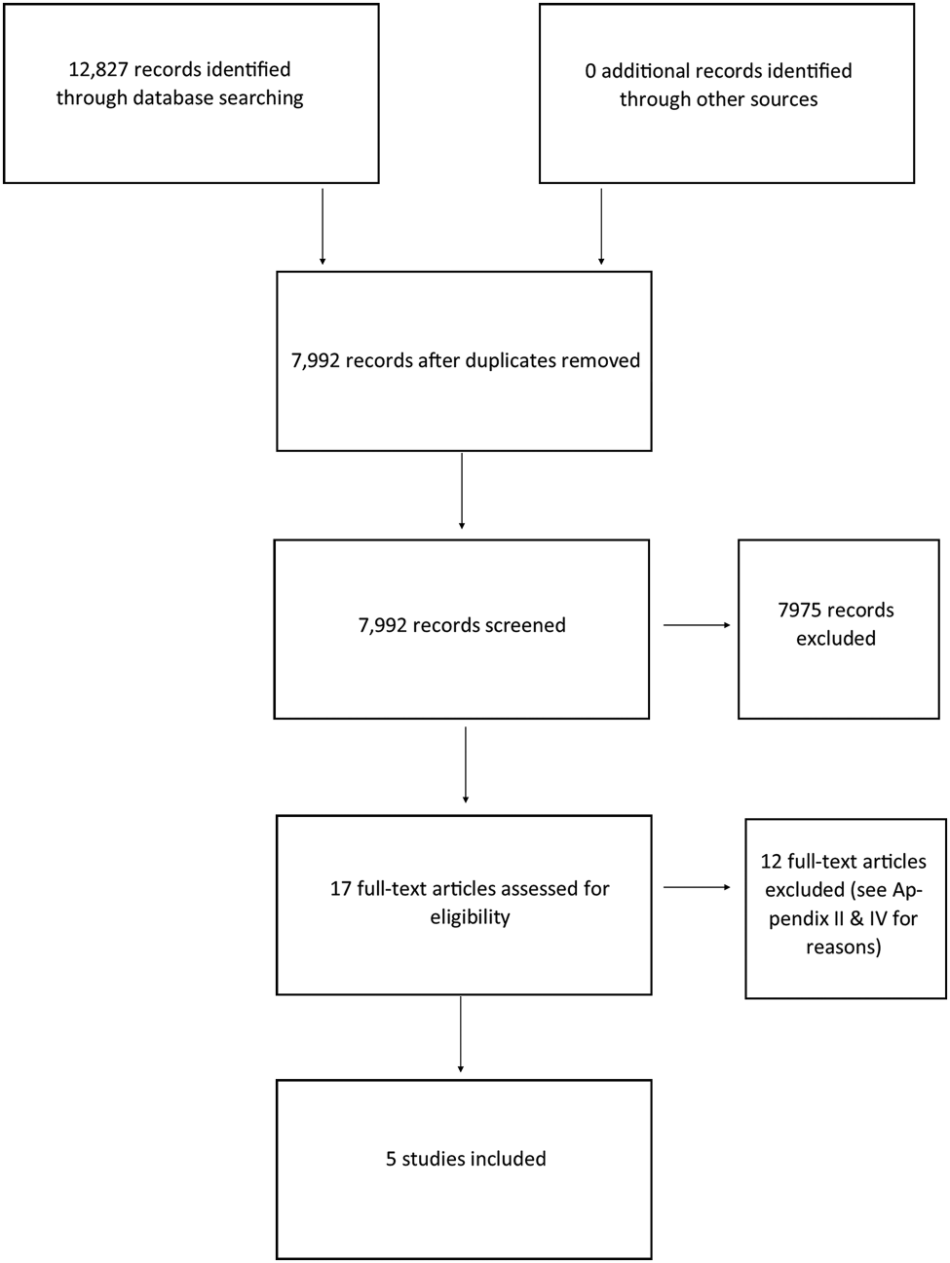
Flow chart showing the study selection process. See online supplementary appendix II for the Characteristics of Included Studies table and online supplementary appendix III for the Characteristics of Excluded Studies table.

We found 7992 reports after removing duplicates and screened all titles and abstracts, and the full texts of 17 potentially relevant studies. Five studies,^25–29^ all uncontrolled before-after studies (without independent comparison groups), were included (see figure 1).

### Risk of bias

Table 1 demonstrates each study's risk of bias.

**Table 1:** Risk of bias ratings for each domain for each study

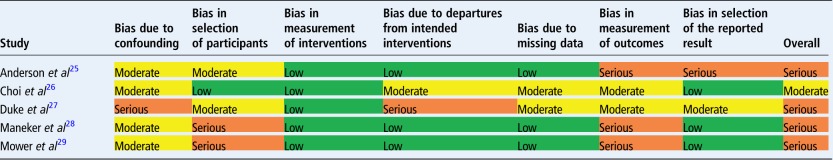

Risk of bias rating is based on a 4-point scale from low to moderate, serious and critical.

### Intervention effects

Only five eligible studies were included, all uncontrolled before-after studies at high risk of bias; our confidence in the effect estimates is therefore limited with a high level of uncertainty.

#### Mortality rates

Duke *et al*,^27^ reported that mortality rates for children with pneumonia at five Papua New Guinea hospitals decreased by 35% after services were reorganised and pulse oximeters, oxygen concentrators and training were introduced. It is not possible to determine how much of this mortality improvement was due to pulse oximetry versus the provision of training, oxygen systems and other changes.

#### Morbidity

Three studies assessed whether pulse oximeters influence physicians’ clinical decision-making. Paediatric physicians assessed children presenting to an ED and decided their treatment before and after obtaining their pulse oximeter results.^25^ ^28^ ^29^ Studies defined hypoxaemia differently and their physicians used different oxygen saturation (SaO_2_) thresholds to indicate necessary treatment. No independent controls were included, which increased the risk of bias, as did the study designs’ accentuation of pulse oximeters’ value in the clinical process by presenting oximeter results to physicians after their initial evaluations.

No studies directly measured morbidity; however, two examined whether pulse oximeters facilitate morbidity measurement (illness severity scores and diagnosis). Anderson *et al*,^25^ asked physicians to record illness severity assessments, on a 5-point scale, for ill children presenting to the paediatric ward, excluding those with minor orthopaedic/surgical injuries, before and after obtaining pulse oximeter results. Physicians changed 53% of children's scores; two-thirds of these scores were reduced. Physicians in Mower *et al*,^29^ changed the diagnoses in 8% of children with SaO_2_ <95% and 0.7% of children with SaO_2_ ≥95% after receiving pulse oximeter results.

#### Length of stay and influence on admission rates

In Choi and Claudius,^26^ the average time spent in a paediatric ED triage when pulse oximeters were used was 17% less than in the same ED a year previously, when pulse oximeters were not used.

Maneker *et al*,^28^ reported that 46/69 children (67%) who had low SaO_2_ (SaO_2_<92%) had not been clinically expected to have low SaO_2_, while 23 (33%) had been expected to have low SaO_2_. After obtaining pulse oximeter results, physicians admitted 13/46 (28%) of children with unexpectedly low SaO_2_ (who would have been discharged without pulse oximetry) and admitted 1/23 (4%) of children who expectedly had low SaO_2_.^28^ Mower *et al*,^29^ found that after receiving pulse oximeter results, physicians admitted 5 additional children of the 305 who had SaO_2_<95% (2%) and 5 additional children of the 1822 who had SaO_2_≥95% (0.3%).^29^

#### Secondary research question

Management plans changed for 19% of children in Anderson *et al*;^25^ most of these plans became less intense. In Maneker *et al*,^28^ management plans changed for 91% of children who unexpectedly had low SaO_2_ (SaO_2_<92%); 90% of these were started on oxygen therapy. Management plans also changed for 43% of children who expectedly had low SaO_2_; 90% of these were started on oxygen. In Mower *et al*,^29^ after receiving pulse oximeter results, physicians ordered new diagnostic tests for 20% of children with SaO_2_<95% and for 0.5% of children with SaO_2_≥95%; they ordered new treatments for 11% of children with SaO_2_<95% and for 1% of children with SaO_2_≥95%.

## Discussion

Pulse oximeters are routinely used in high-income countries, but are implemented without consistent guidelines of when/how to use them and little research on how their routine use impacts health outcomes or resources. New programmes encouraging pulse oximeter use in low-income countries should address these inadequacies in the evidence base and promote evidence-based decision-making.

Only five studies, all before-after studies at high risk of bias, were identified. Potential dissimilarities in patient/location characteristics existed between time periods in two studies;^26^ ^27^ there were no independent controls in three,^25^ ^28^ ^29^ and in these, physicians were perhaps more inclined to respond to pulse oximetry because results were given after, not during, initial evaluations (unlike outside study settings). In the study providing mortality data, oxygen concentrators, training and other improvements were introduced with pulse oximeters;^27^ while this study points to important effects of improving oxygen therapy systems, from identification to management, it provides only indirect information on possible effects of wide-scale pulse oximetry adoption, for example, primary care facilities for guiding referral. Other challenges in generalising the findings are that the studies were conducted in the USA and Papua New Guinea and none were conducted in primary care facilities. Although the data are drawn from US studies and those conducted over 15 years ago, available results have some value as they suggest how pulse oximeter introduction impacts physicians’ decision-making, the key mechanism by which pulse oximetry influences practice.

Study design limitations reduce our confidence in the included studies’ effect estimates. However, there is some evidence to indicate that pulse oximetry may lead to improved health outcomes, with lower mortality rates (when combined with improved/adequate oxygen administration) and reduced time in ED triage; pulse oximetry may change physicians’ decisions regarding illness severity, and increase hospital admissions related to previously unrecognised hypoxaemia (note: hypoxaemia definitions varied from <92% to 95%). Routine pulse oximetry may also influence diagnostic tests and treatments used. Mower *et al*,^29^ argue that physicians generally accurately judged SaO_2_ clinically when very high or low, but made more management changes when moderately low.

It is unclear from the literature how pulse oximetry impacts resource utilisation, even though pulse oximetry campaigns focus on low-income settings, where cost-effectiveness is crucial.

In Maneker *et al*,^28^ and Mower *et al*,^29^ pulse oximetry increased resource use through increased admissions and oxygen therapy for children with otherwise undetected hypoxaemia. Pulse oximetry led to reduced resource utilisation in two studies: in Anderson *et al*^25^ two-thirds of children whose illness severity scores changed were then considered less severely ill, and two-thirds of children whose management plans changed were then managed less aggressively; in Choi and Claudius,^26^ pulse oximetry led to reduced triage time.

Pulse oximetry could facilitate quicker diagnosis, so effective treatment starts earlier and recovery likelihood increases, reducing future resource use. Oximetry can reduce resource waste by indicating when to end treatment, and by decreasing false-positives. However, in Schroeder *et al*,^30^ hospital stays were on average 1.6 days longer because of pulse oximetry as 26% of children met discharge criteria except needing oxygen according to pulse oximeters. If additional treatment was unnecessary (eg, inappropriate thresholds were used), then resources were wasted (as the authors assumed). However, if pulse oximetry enabled detection of hypoxic children who would not otherwise obtain treatment, then the additional resources were justified.

**Figure 2:**
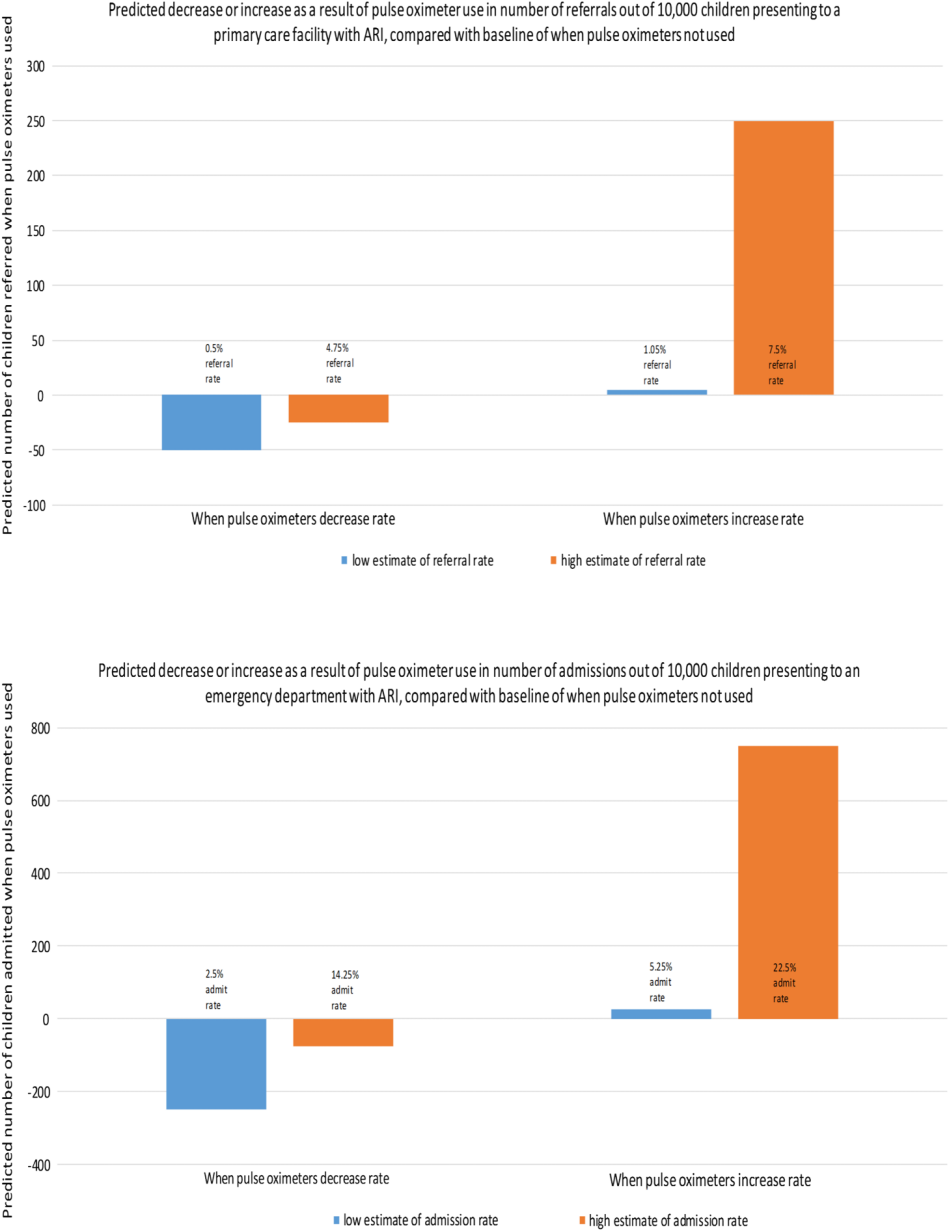
Simple, hypothetical illustration of introducing pulse oximetry into primary care or walk-in clinical settings illustrating the trade-offs that may be apparent in terms of increased or decreased referral or admission rates based on plausible (low and high) estimates of existing rates and true prevalence of hypoxaemia. Note that in low-income countries children often have multiple acute respiratory infections (ARI) episodes per year. The estimates for the baseline (when pulse oximeters are not used) were the following: low estimate for referral rates from primary care facilities: 100 (1%); high estimate: 500 (5%); low estimate for admission from emergency department (ED): 500 (5%); high estimate: 1500 (15%). Referral and admission rates for when pulse oximeters are used were estimated by assuming an increase or decrease in primary care referrals/ED admissions of 5–50% over or below the baseline referral/admission rates. Hypoxaemia prevalence in children aged 7 days–36 months presenting to an ED with ARI has been shown to be as high as 59%^31^ (when hypoxaemia defined as SaO_2_<91%) so these high estimates for referral/admission rates are reasonable. Referral/admission rates would be higher in a population if: the true hypoxaemia prevalence in the population is higher (eg, due to high altitude, seasonal effects on bronchiolitis, predisposing environmental factors for asthma); a larger proportion of hypoxaemic children are being missed by clinical evaluation; or higher thresholds are used to define hypoxaemia. The converse of these conditions would lead to lower referral/admission rates, as would a reduction in the number of false-positives as a result of improved accuracy of hypoxaemia diagnosis over clinical signs.

Although not discussed here, pulse oximetry also has important resource implications in outpatient facilities, where hypoxaemia prevalence in children, while lower than in hospitals, is still considerable (eg, 4–12%),^2^ and where pulse oximetry could facilitate timely recognition of necessary care or referral to hospital. Figure 2 illustrates the range of effects of introducing pulse oximetry.

Randomly assigning health facilities to pulse oximeter introduction (with training) or no pulse oximetry could provide robust data on resource use (admissions, diagnostic tools, treatments, referrals, length of stay), health outcomes (mortality, morbidity, re-presentations), and which thresholds, if any, would be most effective for treatment initiation, if studies were sufficiently large. Such pragmatic studies could be done alongside implementation programmes and could elucidate whether pulse oximetry impacts resource use and health outcomes within cost-effectiveness analyses.

If evidence suggests pulse oximetry increases resource utilisation then health workers, facility managers and public health practitioners would need to weigh cost-benefit trade-offs between using scarce resources on pulse oximetry or on other interventions. Context-specific formal cost-effectiveness analyses could be performed to help address these issues but these are rarely done when technologies are introduced into low-income countries’ health systems. Such research should be independent and transparent evaluations feeding into wider, evidence-based and inclusive processes for decision-making on resource allocation within health systems.

More research is also needed on the best ways to use pulse oximeters, particularly concerning SaO_2_ thresholds.

In high-income settings, disease management guidelines rarely recommend SaO_2_ thresholds for diagnosing, evaluating or monitoring children.^12^ ^13^ When specific SaO_2_ thresholds are recommended, they differ across organisations, even though WHO's 2012 Recommendations for Management of Common Childhood Conditions provide clear guidance that oxygen be administered if SaO_2_<90% (for children at ≤2500 m).^14^ ^15^ Conversely, the Canadian Paediatric Society warns “it is important to recognize that setting arbitrary thresholds for oxygen therapy will influence admission rates”.^16^ Setting thresholds is complicated because pulse oximeter results may not be considered in isolation from clinical findings, SaO_2_ can naturally fluctuate over a day, and studies show that ‘healthy’ SaO_2_ differs by age and altitude.^20–22^ ^32^ A few studies have investigated whether outcomes are comparable when thresholds higher than WHO's <90% are used: Cunningham *et al*,^33^ found cough resolution time in children with bronchiolitis was equivalent when a <94% or <90% threshold was used for oxygen therapy while Lazzerini *et al*,^34^ found that hypoxaemia predicted elevated mortality risk in children with acute lower respiratory infection when a <92% or <90% hypoxaemia threshold was used. It is therefore perhaps unexpected that no studies have examined health system consequences of implementing the WHO guidance of using SaO_2_<90% thresholds.

In absence of clear guidelines, opinion differs on which thresholds should indicate hypoxaemia and prompt admission, referral, oxygen therapy or other treatments. When emergency physicians were surveyed, there was considerable variability in the lowest SaO_2_ for which they would discharge a 2-year-old with pneumonia and a 10-month-old with bronchiolitis.^18^ Maneker *et al*^28^ and Mower *et al*,^29^ defined low SaO_2_ as ≤92% and <95% respectively, thus reducing results comparability.

Threshold choice can substantially impact health system outcomes; thus in Schuh *et al*'s^35^ randomised clinical trial of infants (excluded from this review because all children received pulse oximeter readings), admission rates were sensitive to small saturation differences: 41% of children in the control group were admitted within 72 h versus 25% of children whose displayed SaO_2_s were artificially increased by 3%.

## Conclusions

Pulse oximeters are routinely used in high-income countries and international organisations are investing in programmes to promote pulse oximetry in low-income countries, but there is little evidence, from any region or setting, on the impact or optimal use of pulse oximeters when children present to a health facility. More research is needed on how pulse oximetry impacts health outcomes and services, how knowledge of SaO_2_ should be integrated with other clinical findings, whether defining ‘one-size fits all’ thresholds is possible or even useful, for hypoxaemia and in diagnosing/monitoring specific diseases, and how pulse oximetry affects resource utilisation. Such pragmatic research could accompany pulse oximeter implementation efforts and would provide much needed evidence.[Table ARCHDISCHILD2015309638TB2]

**Table 2 ARCHDISCHILD2015309638TB2:** Summary of findings

Pulse oximeters versus no pulse oximeters to inform diagnosis and treatment (excluding operative surgical care)					
Population: newborns, children and adolescents aged up to 19 years Intervention: pulse oximeter readings Control: populations with no pulse oximeter readings Outcomes: mortality rates, morbidity, length of hospital stay					
** Outcomes**	** Overall outcome difference between control and intervention group**	**Number of participants by outcome (studies)**	** Relative effect (with 95% CI)**	** Absolute effect (with 95% CI)**	**Quality of the evidence—Grade**
Mortality rates	The introduction of pulse oximeters alone may lead to a reduction in mortality rates.^27^	11 291^27^	RR: 0.648 (0.533 to 0.788)	Reduction of 1.75% (1.101 to 2.398) or 17 fewer deaths per 1000 patients	Very low*
Morbidity:	When pulse oximeter results are obtained in the ED, the assessed degree of illness and the diagnosis for children may be different than if pulse oximeter results are not obtained. This is especially the case for children who do not have a diagnosis of ‘well’, ‘minor orthopaedic injuries’ or ‘minor surgical injuries’, and/or is more likely in children who have low SaO_2_ values.^25^ ^29^	2564^25^ ^29^	n/a	n/a	Very low†
Length of hospital stay	The introduction of pulse oximetry into triage may decrease the average time children spend in triage and may increase the proportion of hypoxic children who are admitted.^26^ ^28^ ^29^	622^26^ ^28^ ^29^	Time spent in triage: Mean difference: 50 min (5.405 to 94.595) Proportion of hypoxic children admitted: n/a	Time spent in triage: 17 fewer minutes spent in triage per 100 min Proportion of hypoxic children admitted: n/a	Very low‡
Secondary research question: treatment and management	When pulse oximeter results are obtained in the ED, the management plans for children may be different than if pulse oximeter results are not obtained. This is especially the case for children who do not have a diagnosis of ‘well’, ‘minor orthopaedic injuries’ or ‘minor surgical injuries’, and/or is more likely in children who have low SaO_2_ values, particularly if these are unexpectedly low.^25^ ^28^ ^29^	2633^25^ ^28^ ^29^	n/a	n/a	Very Low§

See online supplementary appendix IV for a more detailed summary of findings table.

*Non-controlled before-after study: Study limitations—there is a high risk of bias as the Duke *et al*^27^ study had a serious risk of bias, due mainly to the fact that oxygen concentrators and training were introduced into the study hospitals concurrently with pulse oximeters so it is not possible to determine how much of the change in mortality rates shown in the study was due specifically to pulse oximeter use; indirectness—the study was looking at the impact of the introduction of pulse oximeters and oxygen concentrators on mortality rates, rather than just the introduction of pulse oximeters alone; imprecision—only one study (and it did not report CIs for the measure of interest); this outcome has therefore been downgraded from Low to Very Low.

†Non-controlled before-after studies: Study limitations—there is a high risk of bias as both of these studies had a serious risk of bias, because the physicians in both studies were aware of the intervention status of the participants and so may have been more likely to take the pulse oximeter results into account than had they received the pulse oximeter results during their initial evaluations; in addition the authors of Mower *et al*^29^ excluded 20% of children who could have been included in the study, potentially affecting the results, and the authors of Anderson *et al*^25^ excluded a subgroup of children from the analyses when it became evident that pulse oximeter results did not impact their management, so the study's results of pulse oximeter impact were exaggerated; indirectness—the changes in degree of illness and diagnosis shown in these studies are not actual changes in morbidity, they are changes in physicians’ perceptions of morbidity; also both studies were looking at different suboutcomes and different subgroups from each other, most of which were not directly relevant to, or only partially relevant to, the review; imprecision—only two studies (neither of which reported any CIs); this outcome has therefore been downgraded from Low to Very Low.

‡Non-controlled before-after studies: Study limitations—there is a high risk of bias as two of the studies had a serious risk of bias, because the physicians in both studies were aware of the intervention status of the participants and so may have been more likely to take the pulse oximeter results into account than had they received the pulse oximeter results during their initial evaluations; in addition 20% and 32% of potential participants were not included in the Mower *et al*^29^ and Maneker *et al*^28^ studies, respectively, potentially affecting the results; indirectness—the outcomes investigated in the three studies (length of stay in emergency department (ED) triage, and % admitted) are indirectly related to but not exactly the same as, the outcome of length of hospital stay; imprecision—only three studies (none of which reported any CIs); this outcome has therefore been downgraded from Low to Very Low.

§Non-controlled before-after studies: Study limitations—there is a high risk of bias as all three of these studies had a serious risk of bias, because the physicians in all three studies were aware of the intervention status of the participants and so may have been more likely to take the pulse oximeter results into account than had they received the pulse oximeter results during their initial evaluations; in addition 20% and 32% of potential participants were not included in the Mower *et al*^29^ and Maneker *et al*^28^ studies, respectively, potentially affecting the results; also the authors of Anderson *et al*^25^ excluded a subgroup of children from the analyses when it became evident that pulse oximeter results did not impact their management, so the study's results of pulse oximeter impact were exaggerated; indirectness—the secondary research question considered the impact of pulse oximeter use on the proportion of children receiving oxygen therapy—only one of the studies actually reported the number of children in both groups who received oxygen therapy while the other two studies only reported results on outcomes that are related to oxygen therapy, by, like oxygen therapy, being examples of treatment and management; also all three studies were looking at different suboutcomes and different subgroups from each other, most of which were not directly relevant to, or only partially relevant to, the review; imprecision—only three studies (none of which reported any CIs); this outcome has therefore been downgraded from Low to Very Low.

## Supplementary Material

Web Appendix I

Web Appendix II

Web Appendix III

Web Appendix IV

## References

[1] World Health Organization. Manual on use of oxygen therapy in children 2014 http://www.who.int/maternal_child_adolescent/documents/child/en/.

[2] Lozano JM Epidemiology of hypoxaemia in children with acute lower respiratory infection [oxygen therapy in children]. Int J Tuberc Lung Dis 2001;5:496–504. 11409574

[3] Djelantik IG, Gessner BD, Sutanto A, et al Case fatality proportions and predictive factors for mortality among children hospitalized with severe pneumonia in a rural developing country setting. J Trop Pediatr 2003;49:327–32. 10.1093/tropej/49.6.327 14725409

[4] Orimadegun A, Ogunbosi B, Orimadegun B Hypoxemia predicts death from severe falciparum malaria among children under 5 years of age in Nigeria: the need for pulse oximetry in case management. Afr Health Sci 2014;14:397–407. 10.4314/ahs.v14i2.16 25320590 PMC4196412

[5] Desalu OO, Onyedum CC, Iseh KR, et al Asthma in Nigeria: are the facilities and resources available to support internationally endorsed standards of care? Health Policy 2011;99:250–4. 10.1016/j.healthpol.2010.10.006 21056506

[6] English M, Gathara D, Mwinga S, et al Adoption of recommended practices and basic technologies in low-income setting. Arch Dis Child 2014;99:452–6. 10.1136/archdischild-2013-305561 24482351 PMC3995214

[7] Lifebox. Value of a lifebox. http://www.lifebox.org/safe-surgery/value-of-a-lifebox/ (accessed May 2015).

[8] Peterson CL, Chen TP, Ansermino M, et al Design and evaluation of a low-cost smartphone pulse oximeter. *Sensors* (Basel) 2013;13:16882–93. 10.3390/s131216882 24322563 PMC3892845

[9] World Health Organization. Global pulse oximetry project: First international consultation meeting. Background document. 2008 http://www.who.int/patientsafety/events/08/1st_pulse_oximetry_meeting_background_doc.pdf (accessed Aug 2015).

[10] Feinmann J Pulse oximeters for all. BMJ 2011;343:d8085 10.1136/bmj.d8085 22171350

[11] Hanning CD, Alexander-Williams JM Fortnightly review: pulse oximetry: a practical review. BMJ 1995;311:367 10.1136/bmj.311.7001.367 7640545 PMC2550433

[12] Bronchiolitis in children: NICE guideline, draft for consultation 2014 http://www.nice.org.uk/guidance/ng9/documents/bronchiolitis-in-children-draft-nice-guideline2 (accessed April 2015).

[13] Guidelines for the management of community acquired pneumonia in adults. British Thoracic Society 2009 https://www.brit-thoracic.org.uk/document-library/clinical-information/pneumonia/adult-pneumonia/a-quick-reference-guide-bts-guidelines-for-the-management-of-community-acquired-pneumonia-in-adults/ (accessed April 2015).

[14] Guidelines for the use of pulse oximeters. NHS Calderdale CCG, NHS Greater Huddersfield CCG, NHS North Kirklees CCG, NHS Wakefield CCG 2013 http://www.greaterhuddersfieldccg.nhs.uk/fileadmin/greaterhuddersfield/Medicines_Management/Pulse_Oximetry_guidelines_181013.pdf (accessed April 2015).

[15] Recommendations for management of common childhood conditions. World Health Organization 2012 http://whqlibdoc.who.int/publications/2012/9789241502825_eng.pdf (accessed Aug 2015). 23720866

[16] Friedman JJ, Rieder MJ, Walton JM Bronchiolitis: recommendations for diagnosis, monitoring and management of children one to 24 months of age. Paediatr Child Health 2014;19:485–91. 25414585 10.1093/pch/19.9.485 PMC4235450

[17] Ralston SL, Lieberthal AS, Meissner HC, et al Clinical practice guideline: the diagnosis, management, and prevention of bronchiolitis. Pediatrics 2014;134:e1474–502. 10.1542/peds.2014-2742 25349312

[18] Brown L, Dannenberg B Pulse oximetry in discharge decision-making: a survey of emergency physicians. CJEM 2002;4:388–93. 17637155 10.1017/s1481803500007880

[19] Ingram G, Munro M The use (or otherwise) of pulse oximetry in general practice. Br J Gen Pract 2005;55:501–2. 16004733 PMC1472779

[20] Fouzas S, Priftis KN, Anthracopoulos MB Pulse oximetry in pediatric practice. Pediatrics 2011;128:740–52. 10.1542/peds.2011-0271 21930554

[21] Schult S, Canelo-Aybar C Oxygen saturation in healthy children aged 5 to 16 years residing in Huayllay, Peru at 4340m. High Alt Med Biol 2011;12:89–92. 10.1089/ham.2009.1094 21452970 PMC3114159

[22] Subhi R, Smith K, Duke T When should oxygen be given to children at high altitude? A systematic review to define altitude-specific hypoxaemia. Arch Dis Child 2009;94:6–10. 10.1136/adc.2008.138362 18829620

[23] 13a good practice data extraction form. EPOC resources for review authors. Oslo: Norwegian Knowledge Centre for the Health Services: Effective Practice and Organisation of Care (EPOC). 2014 https://epocoslo.cochrane.org/epoc-specific-resources-review-authors (accessed April 2015).

[24] Sterne JAC, Higgins JPT, Reeves BC, on behalf of the development group for ACROBAT-NRSI. A Cochrane risk of bias assessment tool: For non-randomized studies of interventions (ACROBAT-NRSI). 2014 https://sites.google.com/site/riskofbiastool/ (accessed Apr 2015).

[25] Anderson AB, Zwerdling RG, Dewitt TG The clinical utility of pulse oximetry in the pediatric emergency department setting. Pediatr Emerg Care 1991;7:263–6. 10.1097/00006565-199110000-00001 1754483

[26] Choi J, Claudius I Decrease in emergency department length of stay as a result of triage pulse oximetry. Pediatr Emerg Care 2006;22:412–14. 10.1097/01.pec.0000221340.26873.2f 16801841

[27] Duke T, Wandi F, Jonathan M, et al Improved oxygen systems for childhood pneumonia: a multihospital effectiveness study in Papua New Guinea. Lancet 2008;372:1328–33. 10.1016/S0140-6736(08)61164-2 18708248

[28] Maneker AJ, Petrack EM, Krug SE Contribution of routine pulse oximetry to evaluation and management of patients with respiratory illness in a pediatric emergency department. Ann Emerg Med 1995;25:36–40. 7802367 10.1016/s0196-0644(95)70352-7

[29] Mower WR, Sachs C, Nicklin EL, et al Pulse oximetry as a fifth pediatric vital sign. Pediatrics 1997;99:681–6. 10.1542/peds.99.5.681 9113944

[30] Schroeder AR, Marmor AK, Pantell RH, et al Impact of pulse oximetry and oxygen therapy on length of stay in bronchiolitis hospitalizations. Arch Pediatr Adolesc Med 2004;158:527–30. 10.1001/archpedi.158.6.527 15184214

[31] Onyango FE, Steinhoff MC, Wafula EM, et al Hypoxaemia in young Kenyan children with acute lower respiratory infection. BMJ 1993; 306:612–15. 10.1136/bmj.306.6878.612 8369033 PMC1676956

[32] Vargas MH, Heyaime-Lalane J, Perez-Rodriguez L, et al Day-night fluctuation of pulse oximetry: an exploratory study in pediatric inpatients. Rev Invest Clin 2008;60:303–10. 18956552

[33] Cunningham S, Rodriguez A, Adams T, et al Oxygen saturation targets in infants with bronchiolitis (BIDS): a double-blind, randomised, equivalence trial. Lancet 2015;386:1041–8. 10.1016/S0140-6736(15)00163-4 26382998 PMC4673090

[34] Lazzerini M, Sonego M, Pellegrin MC Hypoxaemia as a mortality risk factor in acute lower respiratory infections in children in low and middle-income countries: systematic review and meta-analysis. PloS ONE 2015; 10:e0136166 10.1371/journal.pone.0136166 26372640 PMC4570717

[35] Schuh S, Freedman S, Coates A, et al Effect of oximetry on hospitalization in bronchiolitis: a randomized clinical trial. JAMA 2014;312:712–18. 10.1001/jama.2014.8637 25138332

