# Supplementary material for: Does pulse oximeter use impact health outcomes? A systematic review
Source: Arch Dis Child. 2015 Dec 23;101(8):694–700. doi: 10.1136/archdischild-2015-309638 (PMC4975806; doi:10.1136/archdischild-2015-309638)
Supplement: Web Appendix I [file archdischild-2015-309638-s1.pdf]

Appendix I: search terms and search methods

Database	Searches performed	Date	Notes
Dare	**1. [any field] “pulse oximeter” OR [any field] “pulse oximetry”**	January 7, 2015	The Dare tickbox was ticked for the search
Pubmed	1. (((“Infant”[Mesh]) OR “Child”[Mesh]) OR “Adolescent”[Mesh]) OR “Pediatrics”[Mesh]<br>2. (Newborn*[Title/Abstract] OR Neonat*[Title/Abstract] OR Baby*[Title/Abstract] OR Babies[Title/Abstract] OR Infant*[Title/Abstract] OR Child*[Title/Abstract] OR Kid*[Title/Abstract] OR Toddler*[Title/Abstract] OR Adoles*[Title/Abstract] OR Teen*[Title/Abstract] OR Boy*[Title/Abstract] OR Girl*[Title/Abstract] OR Paediatric*[Title/Abstract] OR Peadiatric*[Title/Abstract] OR Pediatric*[Title/Abstract])<br>3. #1 OR #2<br>4. “Oximetry”[Mesh]<br>5. (“Pulse oximeter*”[Title/Abstract] OR “Pulse oximetry”[Title/Abstract])<br>6. #4 OR #5<br>**7. #3 AND #6**	January 9, 2015	
Web of Science	1. TS=(“Pulse oximeter*” OR “Pulse oximetry”) OR TI=(“Pulse oximeter*” OR “Pulse oximetry”)<br>2. TS=(Newborn* OR Neonat* OR Baby* OR Babies OR Infant* OR Child* OR Kid* OR Toddler* OR Adoles* OR Teen* OR Boy* OR Girl* OR Paediatric* OR Peadiatric* OR Pediatric*) OR TI=(Newborn* OR Neonat* OR Baby* OR Babies OR Infant* OR Child* OR Kid* OR Toddler* OR Adoles* OR Teen* OR Boy* OR Girl* OR Paediatric* OR Peadiatric* OR Pediatric*)<br>**3. #1 AND #2**	January 13, 2015	In “All databases” and for all years
Cochrane library	**1. “pulse oximeter” OR “pulse oximetry”**	January 8, 2015	In “Title, Abstract or Keywords”<br>“Cochrane reviews”, “Other reviews” and

			"Technology assessments" tick boxes were ticked
Medion	**1. Pulse oximeter**<br>**2. Pulse oximetry**	January 8, 2015	In "Topics"
WHO Global Health Library	**1. Pulse oximeter**<br>**2. Pulse oximeter**<br>**3. Pulse oximetry**<br>**4. Pulse oximetry**	January 8, 2015	#1 and #3 were in "title" and in "regional"; #2 and #4 were in "subject" and in "regional"
Embase	1. Pulse oximeter/ or pulse oximetry/<br>2. ("Pulse oximeter*" or "Pulse oximetry").mp.<br>3. ("Pulse oximeter*" or "Pulse oximetry").m_titl.<br>4. #1 OR #2 OR #3<br>5. (Newborn* OR Neonat* OR Baby* OR Babies OR Infant* OR Child* OR Kid* OR Toddler* OR Adoles* OR Teen* OR Boy* OR Girl* OR Paediatric* OR Peadiatric* OR Pediatric*).mp.<br>6. (Newborn* OR Neonat* OR Baby* OR Babies OR Infant* OR Child* OR Kid* OR Toddler* OR Adoles* OR Teen* OR Boy* OR Girl* OR Paediatric* OR Peadiatric* OR Pediatric*).m_titl.<br>7. #5 OR #6<br>**8. #4 AND #7**	January 14, 2015	-#1 was done by "mapping to subject headings" but not ticking "explode"<br>-mp=title, abstract, subject headings, heading word, drug trade name, original title, device manufacturer, drug manufacturer, device trade name, keyword
Global Health	1. ("Pulse oximeter*" OR "Pulse oximetry").mp.<br>2. ("Pulse oximeter*" OR "Pulse oximetry").m_titl.<br>3. #1 or #2<br>4. (Newborn* OR Neonat* OR Baby* OR Babies OR Infant* OR Child* OR Kid* OR Toddler* OR Adoles* OR Teen* OR Boy* OR Girl* OR Paediatric* OR Peadiatric* OR Pediatric*).mp.<br>5. (Newborn* OR Neonat* OR Baby* OR Babies OR Infant* OR Child* OR Kid* OR Toddler* OR Adoles* OR Teen* OR Boy* OR Girl* OR Paediatric* OR Peadiatric* OR Pediatric*).m_titl.<br>6. #4 OR #5	January 14, 2015	mp=abstract, title, original title, broad terms, heading words, identifiers, cabicodes

	**7. #3 AND #6**		
CINAHL	1. TI ("Pulse oximeter*" OR "Pulse oximetry") OR AB ("Pulse oximeter*" OR "Pulse oximetry")<br><br>2. TI (Newborn* OR Neonat* OR Baby* OR Babies OR Infant* OR Child* OR Kid* OR Toddler* OR Adoles* OR Teen* OR Boy* OR Girl* OR Paediatric* OR Peadiatric* OR Pediatric*) OR AB (Newborn* OR Neonat* OR Baby* OR Babies OR Infant* OR Child* OR Kid* OR Toddler* OR Adoles* OR Teen* OR Boy* OR Girl* OR Paediatric* OR Peadiatric* OR Pediatric*)<br><br>**3. #1 AND #2**	January 14, 2015	

Note: the search(es) shown in **bold** is/are the one(s) from which results were taken

The websites of the following organizations were searched using the search terms 'pulse oximeter' and 'pulse oximetry' to obtain unpublished reports: the World Health Organization, the World Bank, USAID, Public Health England, the UK's Department of Health, NHS Evidence – NICE, PATH, Save the Children, Save the Children UK, MSF, Oxfam, UNICEF, the International Union Against Tuberculosis and Lung Disease, the British Lung Foundation, and the World Heart Foundation.
