# Supplementary material for: Does pulse oximeter use impact health outcomes? A systematic review
Source: Arch Dis Child. 2015 Dec 23;101(8):694–700. doi: 10.1136/archdischild-2015-309638 (PMC4975806; doi:10.1136/archdischild-2015-309638)
Supplement: Web Appendix II [file archdischild-2015-309638-s2.pdf]

Appendix II: Characteristics of Included Studies table

Study	Methods	Participants	Intervention	Outcomes
Anderson et.al.,1991	Non-controlled before-after study; illness severity score and management plan recorded before and after the physician obtained the child's pulse oximeter results	437 children who were all in the control group and then the intervention group; average age of 5 years and age range of 1 day to 17 years; Massachusetts, USA	Pulse oximeter results shown to physician after they had decided on illness severity score and management plan	-# and % whose illness severity score changed, and whether considered more vs. less ill after<br>-# and % whose management plans changed, and whether more vs. less aggressive plan after
Choi & Claudius 2006	Non-controlled before-after study; time spent in ED triage was measured before and after pulse oximeters were introduced as a standard triage tool	159 in control group, 89 in intervention group; average age of 11 months and 8 respectively; Los Angeles, USA	Pulse oximeters were introduced into ED triage standard methods	Time spent in ED triage
Duke et.al.,2008	Non-controlled before-after study; mortality rates of children with pneumonia at 5 hospitals before and after pulse oximeters and oxygen concentrators were introduced	7161 in control group, 4130 in intervention group; children up to 5 years old; Papua New Guinea	Pulse oximeters and oxygen concentrators were introduced into 5 hospitals with training	Mortality rates
Maneker et.al.,1995	Non-controlled before-after study; management plan recorded before and after the physician obtained the child's pulse oximeter results	368 children who were all in the control group and then the intervention group; 16% were less than than 6 months old, 59% were 7 to 36 months old, 15% were 37 to 96 months old, and	Pulse oximeter results shown to physician after they had decided on management plan	-# and % of those with unexpectedly low SaO_2 whose management plans changed, including whether oxygen therapy was added, and/or if they were newly admitted

		10% were more than 96 months old; Ohio, USA		-# and % of those with expectedly low SaO2 whose management plans changed, including whether oxygen therapy was added, and/or if they were newly admitted
Mower et.al.,1997	Non-controlled before-after study; management plan recorded before and after the physician obtained the child's pulse oximeter results	2127 children who were all in the control group and then the intervention group; ages ranged from birth to 17 years; Los Angeles, USA	Pulse oximeter results shown to physician after they had decided on management plan	-# and % of those with each oxygen saturation value who obtained new diagnostic tests, new treatments, new diagnoses, and/or were newly admitted
