# Supplementary material for: Does pulse oximeter use impact health outcomes? A systematic review
Source: Arch Dis Child. 2015 Dec 23;101(8):694–700. doi: 10.1136/archdischild-2015-309638 (PMC4975806; doi:10.1136/archdischild-2015-309638)
Supplement: Web Appendix III [file archdischild-2015-309638-s3.pdf]

Appendix III: Characteristics of Excluded Studies table

**Study**	**Reason(s) for exclusion**
Schroeder et.al.,2004	-retrospective case series<br>-all children received pulse oximeter readings<br>-pulse oximeter readings were not necessarily taken at admittance – they may instead have been used for monitoring later as part of treatment
Schuh et.al.,2014	-all children received pulse oximeter readings
Cunningham et.al,2015	-all children received pulse oximeter readings
