# Supplementary material for: Does pulse oximeter use impact health outcomes? A systematic review
Source: Arch Dis Child. 2015 Dec 23;101(8):694–700. doi: 10.1136/archdischild-2015-309638 (PMC4975806; doi:10.1136/archdischild-2015-309638)
Supplement: Web Appendix IV [file archdischild-2015-309638-s4.pdf]

Appendix IV: detailed summary of findings table

Pulse oximeters vs. no pulse oximeters to inform diagnosis and treatment (excluding operative surgical care)							
Population: newborns, children and adolescents aged up to 19 years<br>Intervention: pulse oximeter readings<br>Control: populations with no pulse oximeter readings<br>Outcomes: mortality rates, morbidity, length of hospital stay							
**Outcomes**	**Overall outcome difference between control and intervention group**	**Number of participants by outcome (studies)**	**Specific study differences between control and intervention group [see Risk of Bias table for risk of bias assessments for each study]**	**Number of participants by study**	**Relative effect (with 95% CI)**	**Absolute effect (with 95% CI)**	**Quality of the evidence - GRADE**
**Mortality rates**	The introduction of pulse oximeters alone may lead to a reduction in mortality rates.[27]	11,291 (1 – Duke et.al., 2008)	-Mortality rate changed from 4.97% to 3.22% (35% relative reduction) [for those admitted with a diagnosis of pneumonia] after pulse oximeters, oxygen concentrators and training introduced[27]<br><br>-Mortality rate changed from 5.53% to 4.1% (26% relative reduction) [for those > 1 month old admitted with any diagnosis] after pulse oximeters, oxygen concentrators and training introduced[27]	11,291<br><br>32,335	RR: 0.648 (0.533, 0.788)	Reduction of 1.75% (1.101, 2.398) or 17 fewer deaths per 1000 patients	Very low[i]
**Morbidity**:<br><br>-Assessed degree of illness	 <br>-When pulse oximeter results are obtained in the ED, the assessed degree of illness and the	2564 (2 – Anderson et.al., 1991; Mower et.al., 1997)	 <br>-No difference [in children with diagnosis of 'well', 'minor orthopaedic injuries' or 'minor surgical injuries'] after physicians received pulse oximeter results[25]	 <br>83	n/a	n/a	Very low[ii]

-Diagnosis	diagnosis for children may be different than if pulse oximeter results are not obtained. This is especially the case for children who do not have a diagnosis of ‘well’, ‘minor orthopaedic injuries’ or ‘minor surgical injuries’, and/or is more likely in children who have low SaO2 values.[25,29]		-53% [of children with diagnoses that were not ‘well’, ‘minor orthopaedic injuries’ or ‘minor surgical injuries’] had a change after physicians received pulse oximeter results; 25% of these were assessed as more ill; 69% were assessed as less ill; direction of change was unknown for 6%[25]<br><br>-diagnosis was changed for 8% of children [of those with SaO2<95%] after physicians received pulse oximeter results [29]<br><br>-diagnosis was changed for 0.7% of children [of those with SaO2≥95%] after physicians received pulse oximeter results [29]	354<br><br>305<br><br>1822			
**Length of hospital stay**	The introduction of pulse oximetry into triage may decrease the average time	622 (3 – Choi & Claudius, 2006; Maneker	-Time spent in ED triage decreased from 4 hours 59 minutes to 4 hours 9 minutes (50 minutes less; a 17% decrease) after pulse oximeters	248	Mean difference: 50 minutes (5.405, 94.595)	17 fewer minutes spent in triage per	Very low[iii]

	children spend in triage and may increase the proportion of hypoxic children who are admitted.[26,28, 29]	et.al., 1995; Mower et.al., 1997)	introduced into emergency department triage[26]<br><br>-28% were admitted only after the pulse oximeter readings were revealed [out of children with unexpectedly low SaO2 (where low SaO2 defined as <92%)][28]<br><br>-4% were admitted only after the pulse oximeter readings were revealed [out of children with expectedly low SaO2 (where low SaO2 defined as <92%)][28]<br><br>-2% were admitted only after the pulse oximeter readings were revealed [out of the children with SaO2<95%][29]<br><br>-0.3% were admitted only after the pulse oximeter readings were revealed [out of the children with SaO2≥95%][29]	 <br>46<br><br>23<br><br>305<br><br>1822	/ n/a	100 minutes / n/a	
**Secondary research question: treatment and management**	When pulse oximeter results are obtained in the ED, the management plans for children may be different than if pulse oximeter results are not obtained.	2633 (3 – Anderson et.al., 1991; Maneker et.al., 1995; Mower et.al., 1997)	-No difference [in children with diagnosis of 'well', 'minor orthopaedic injuries' or 'minor surgical injuries'] after pulse oximeter results received[25]<br><br>-19% [of children with diagnoses that were not 'well', 'minor orthopaedic injuries' or 'minor surgical injuries'] had a change after physicians received	83<br><br>354	n/a	n/a	Very Low[iv]

	This is especially the case for children who do not have a diagnosis of 'well', 'minor orthopaedic injuries' or 'minor surgical injuries', and/or is more likely in children who have low SaO2 values, particularly if these are unexpectedly low.[25,28,29]		pulse oximeter results; 39% of these had more aggressive management after; 58% were managed less aggressively after; direction of change was not documented for 3%[25]					
			-91% [of those who unexpectedly had low SaO2 (where low SaO2 defined as <92%)] had a change after physicians received pulse oximeter results; 90% of these had oxygen added[28]	46				
			-43% [of those who expectedly had low SaO2 (where low SaO2 defined as <92%)] had a change after physicians received pulse oximeter results; 90% of these had oxygen added[28]	23				
			-new diagnostic tests were ordered for 20% [of those with SaO2<95%] after physicians received pulse oximeter results [29]	305				
			-new diagnostic tests were ordered for 0.5% [of those with SaO2≥95%] after physicians received pulse oximeter results [29]	1822				
			-new treatments were ordered for 11% [of those with SaO2<95%] after physicians received pulse oximeter results [29]	305				

			-new treatments were ordered for 1% [of those with SaO2≥95%] after physicians received pulse oximeter results [29]	1822			

Footnotes:

[i] Non-controlled before-after study: Study limitations – there is a high risk of bias as the Duke et.al.,2008 study had a serious risk of bias, due mainly to the fact that oxygen concentrators and training were introduced into the study hospitals concurrently with pulse oximeters so it is not possible to determine how much of the change in mortality rates shown in the study was due specifically to pulse oximeter use; indirectness – the study was looking at the impact of the introduction of pulse oximeters and oxygen concentrators on mortality rates, rather than just the introduction of pulse oximeters alone; imprecision - only 1 study (and it did not report confidence intervals for the measure of interest); this outcome has therefore been downgraded from Low to Very Low.

[ii] Non-controlled before-after studies: Study limitations – there is a high risk of bias as both of these studies had a serious risk of bias, because the physicians in both studies were aware of the intervention status of the participants and so may have been more likely to take the pulse oximeter results into account than had they received the pulse oximeter results during their initial evaluations; in addition the authors of Mower et.al. 1997 excluded 20% of children who could have been included in the study, potentially affecting the results, and the authors of Anderson et.al. 1991 excluded a subgroup of children from the analyses when it became evident that pulse oximeter results did not impact their management, so the study's results of pulse oximeter impact were exaggerated; indirectness – the changes in degree of illness and diagnosis shown in these studies are not actual changes in morbidity, they are changes in physicians' perceptions of morbidity; also both studies were looking at different sub-outcomes and different subgroups from each other, most of which were not directly relevant to, or only partially relevant to, the review; imprecision – only 2 studies (neither of which reported any confidence intervals); this outcome has therefore been downgraded from Low to Very Low.

[iii] Non-controlled before-after studies: Study limitations – there is a high risk of bias as two of the studies had a serious risk of bias, because the physicians in both studies were aware of the intervention status of the participants and so may have been more likely to take the pulse oximeter results into account than had they received the pulse oximeter results during their initial evaluations; in addition 20% and 32% of potential participants were not included in the Mower et.al. 1997 and Maneker et.al. 1994 studies respectively, potentially affecting the results; indirectness – the outcomes investigated in the three studies (length of stay in ED triage, and % admitted) are indirectly related to but not exactly the same as, the outcome of length of hospital stay; imprecision – only 3 studies (none of which reported any confidence intervals); this outcome has therefore been downgraded from Low to Very Low.

[iv] Non-controlled before-after studies: Study limitations - there is a high risk of bias as all three of these studies had a serious risk of bias, because the physicians in all three studies were aware of the intervention status of the participants and so may have been more likely to take the pulse oximeter results into account than had they received the pulse oximeter results during their initial evaluations; in addition 20% and 32% of potential participants were not included in the Mower et.al. 1997 and Maneker et.al. 1994 studies respectively, potentially affecting the results; also the authors of Anderson et.al. 1991 excluded a subgroup of children from the analyses when it became evident that pulse oximeter results did not impact their management, so the study's results of pulse oximeter impact were exaggerated; indirectness – the secondary research question considered the impact of pulse oximeter use on the proportion of children receiving oxygen therapy – only one of the studies actually reported the number of children in both groups who received oxygen therapy while the other two studies only reported results on outcomes that are related to oxygen therapy, by, like oxygen therapy, being examples of treatment and management; also all three studies were looking at different sub-outcomes and different subgroups from each other, most of which were not directly relevant to, or only partially relevant to, the review; imprecision – only 3 studies (none of which reported any confidence intervals); this outcome has therefore been downgraded from Low to Very Low.
